# Executive Summary - Guideline on Telecardiology in the Care of Patients with Acute Coronary Syndrome and Other Cardiac Diseases

**DOI:** 10.5935/abc.20150104

**Published:** 2015-08

**Authors:** Mucio Tavares de Oliveira Jr., Leonardo Jorge Cordeiro de Paula, Milena Soriano Marcolino, Manoel Fernandes Canesin

**Affiliations:** 1 Instituto do Coração, HCFMUSP, São Paulo, SP - Brazil; 2 Universidade Federal de Minas Gerais, Belo Horizonte, MG - Brazil; 3 Universidade Estadual de Londrina, Londrina, PR - Brazil

**Keywords:** Heart Failure / mortality, Myocardial Infarction / mortality, Telemedicine / utilization, Electrocardiography, Mobile Health Units

Cardiology is a very promising field in telemedicine. The transmission of electrocardiograms (ECG) from remote health services or ambulances to a central for analysis is already routine in the approach to acute coronary syndromes (ACS). This approach allows the obtention of expert guidance and referral to appropriate health units, with the potential of saving lives. This impact may be seen in acute myocardial infarction (MI), in which telemedicine has reduced intra-hospital mortality rates from 12.3% to 7.1%^1-4^.

## Basic concepts

In a health system geographically distributed like the Brazilian system, in which Basic Health Units (*Unidades Básicas de Saúde*, UBSs), Emergency Care Units (*Unidades de Pronto Atendimento*, UPAs), secondary hospitals, and ambulances are scattered throughout the country (often in remote locations), and specialized centers are located in advanced care units in large cities (such as tertiary hospitals), telemedicine offers the opportunity to improve the treatment of emergencies. The clinical ability of specialists in tertiary hospitals may be used to improve the care in Remote Care Units (*Unidades Remotas de Atendimento*, URAs), offering support for early diagnosis and therapy guidance for non‑specialist medical practitioners providing medical care to patients in URAs^5,6^.

Communication channels in telemedicine include telephone lines for voice communication and connection to the internet, and for transmission of test results, ECG tracings, and images. Optionally, a video link may be used for visualization of the patient.

### Telemedicine in the approach to ACS^7,8^

**Situation A**: A patient goes by himself to the nearest URA, or calls the prehospital care service and is taken to the URA in a standard ambulance without an electrocardiograph.

The professionals at the URA take the clinical history, examine, and obtain serial ECGs from the patient. The ECG tracings are transmitted along with the clinical history to the telecardiology hub where they are interpreted by cardiologists who quickly prepare and send a report, and guide the professionals at the URA on the appropriate therapy.

**Situation B**: A patient connects with the prehospital care service and an ambulance with an electrocardiograph and without a physician answers the call. Based on the patient's history and interpretation of the ECG, if the cardiologist at the telecardiology hub diagnoses the patient as having an ST‑segment elevation MI (STEMI), he guides the medical team to administer the standard therapy (for example, aspirin and other medications) and transport the patient to a hospital that offers percutaneous coronary intervention (PCI) or to administer fibrinolytic treatment. Even if the diagnosis of STEMI is excluded, the ambulance team is oriented to follow the cardiologist’s instructions about the path to be followed for that patient.

**Situation C**: A patient calls the prehospital care service, an ambulance with a physician and an electrocardiograph answers the call, and the team obtains an ECG that is transmitted to the telecardiology hub. Based on the clinical history and interpretation of the ECG, if the cardiologist at the telecardiology hub determines that the patient has STEMI, he guides the physician to administer treatment for STEMI, such as antiplatelet and anticoagulant agents, and to follow one of these options:

- If the STEMI patient can be transported to a hospital with PCI capability and the PCI can be performed within 120 minutes, or if the patient has contraindication to fibrinolytic treatment, the patient must be transported to the hospital with PCI. The ambulance physician also alerts the hospital to prepare the catheterization laboratory to treat a STEMI patient with primary PCI.
- If the PCI cannot be performed within 120 minutes, the ambulance physician is instructed to first administer fibrinolytic agents, preferably within 30 minutes, and then transport the patient to the nearest hospital equipped with a catheterization laboratory to continue the therapy.
- If the cardiologist in the telecardiology hub confirms that STEMI is not the diagnosis and the ambulance physician determines that the patient has ACS, after receiving the initial therapy the patient should be transferred preferably to a hospital equipped with a catheterization laboratory. If that is not possible, the patient should be transferred to the nearest hospital equipped with an intensive cardiac care unit. If the cardiologist determines that the chest pain protocol should be initiated for the patient, he or she may direct the ambulance team to transport the patient to the nearest hospital, even if the hospital is not equipped with a catheterization laboratory, for monitoring of clinical parameters, ECG, and markers of myocardial necrosis (Figure 1).
  **Figure 1:**
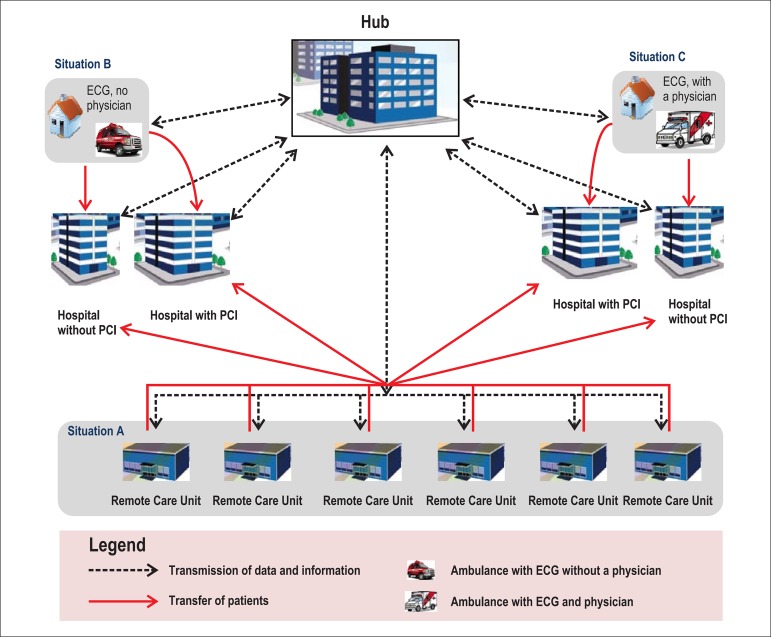
Schematic representation of telemedicine for acute emergency therapy. Treatment strategies using telemedicine are shown for acute coronary syndrome (ACS). ECG: surface electrocardiogram.

**Figure 2:**
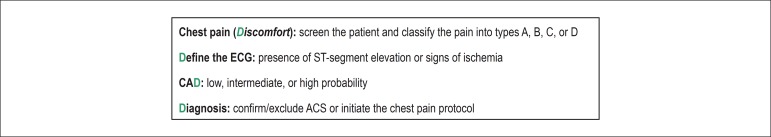
Care systematization for the establishment of the diagnosis in patients with chest pain. ECG: electrocardiogram; CAD: coronary artery disease; ACS: acute coronary syndrome.

To ensure that the transmitted information has good quality and the interaction is valuable, the patient with chest pain should receive a systematic approach, which can be achieved with several methodologies. One of these methodologies takes into account the "4D" for systematization of the diagnosis of ACS (Figure 2)^9^:

- First "**D**": classify the chest pain (***d**iscomfort*) into types A (definitely anginal), B (probably anginal), C (probably not anginal), or D (definitely not anginal).
- Second "**D**": **d**efine whether an ST-segment elevation is present or not in the ECG.
- Third "**D**": if the ECG does not show signs of ischemia, assess the probability of the patient having coronary artery disease (CA**D**) based on the presence of risk factors: age (above 45 years in men and 55 years in women), smoking, diabetes, hypertension, and family history of early CAD (below the age of 55 years in men and 65 years in women).
- Fourth "**D**": the **d**iagnosis of ACS must be confirmed or excluded, or the chest pain protocol should be initiated.

**Figure 3:**
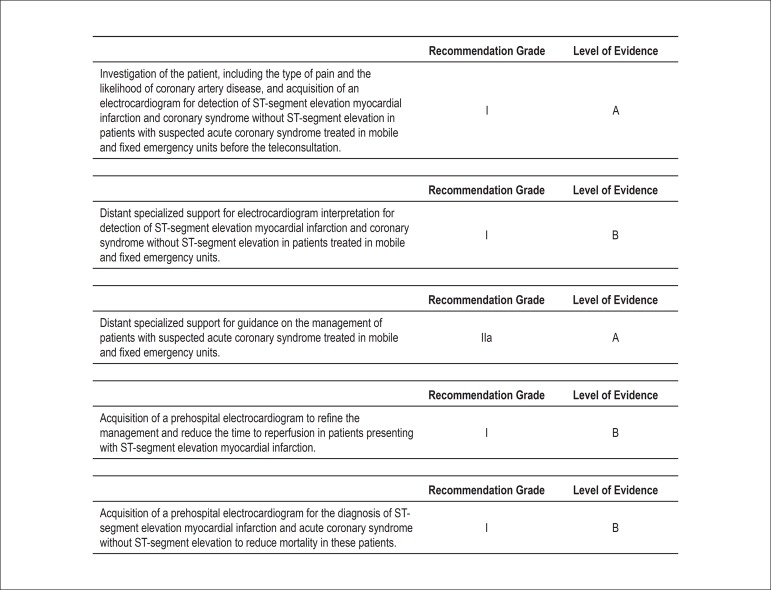
Recommendation grades and levels of evidence of the procedures for management of patients with ACS.

Requirements in telemedicine for adequate diagnosis and treatment of ACS and other acute cardiac diseases (Figure 3)^10^.

**Figure 4:**
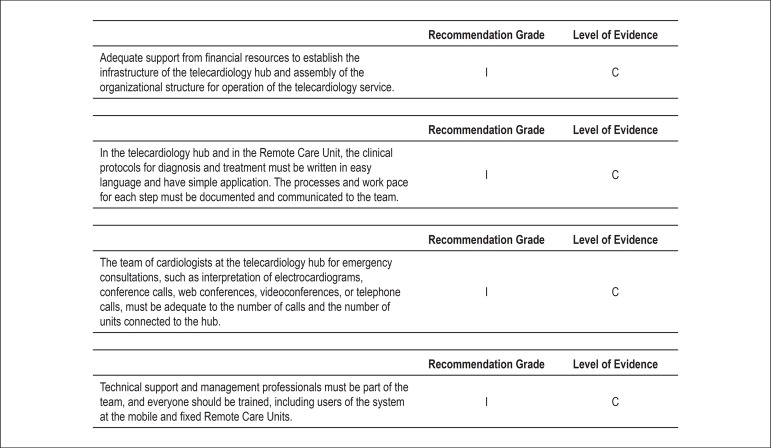
Recommendation grades and levels of evidence for the financial requirements, procedures, and clinical and team protocols in telemedicine for adequate diagnosis and treatment of ACS and other acute cardiac diseases.

Financial requirements, procedures, and clinical and team protocols for deployment of telemedicine for adequate diagnosis and treatment of ACS and other acute cardiac diseases (Figure 4)^10^.

**Figure 5:**
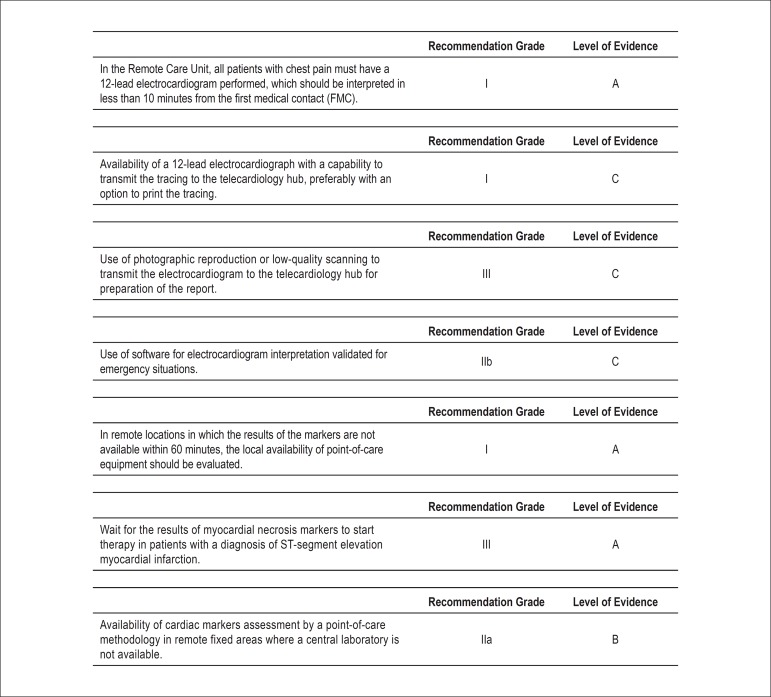
Recommendation grades and levels of evidence for the medical equipment in telemedicine for adequate diagnosis and treatment of ACS and other acute cardiac diseases.

**Figure 6:**
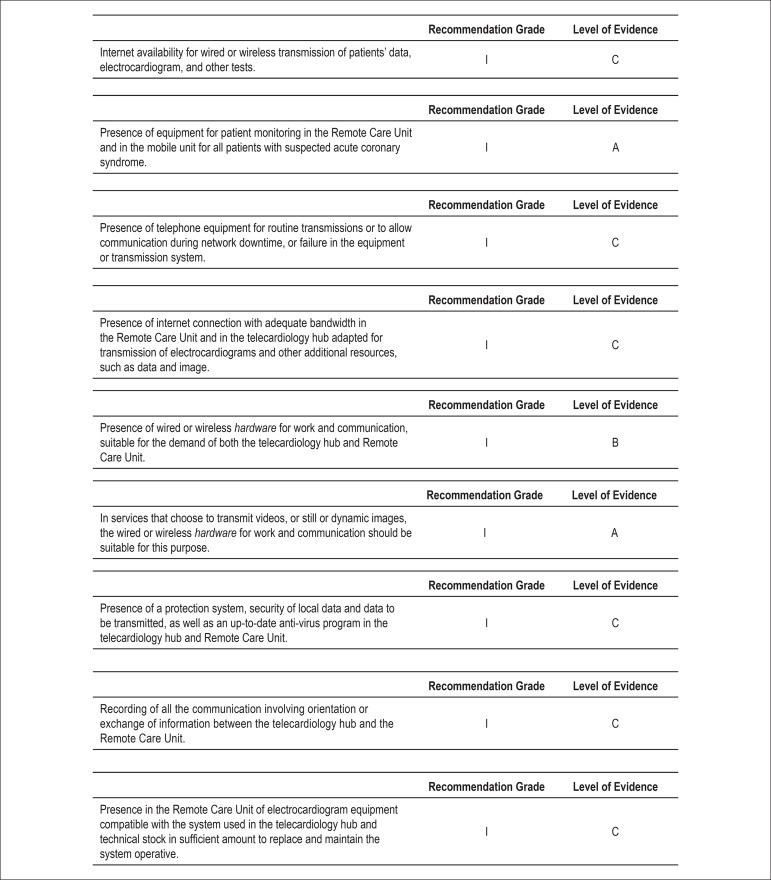
Recommendation grades and levels of evidence for the information technology equipment and services in telemedicine for adequate diagnosis and treatment of ACS and other acute cardiac diseases.

Medical equipment, information technology, and services (Figures 5 and 6)^10^.

### Telecardiology in remote routine diagnosis

One of the most common applications of telecardiology in remote areas is in the analysis of diagnostic tests, such as ECG, Holter, ambulatory monitoring of blood pressure (AMBP), and echocardiography. Other applications include synchronous or asynchronous teleconsulting systems or second opinions, teleauscultation, remote monitoring of blood pressure, vital signs and implantable electronic devices, and educational activities. In addition, telecardiology has important applications in the penitentiary system, in pediatrics, and in fetal cardiology.

### Cardiac arrhythmias and syncope^11-13^

Since several types of cardiac arrhythmia occur in short and unexpected episodes, its diagnosis depends on an ECG recorded during the paroxysmal episode. A standard 10-second surface ECG may not be able to detect the abnormality in the heart rhythm. In this case, long-term monitoring is recommended, such as 24-hour Holter monitoring or event recording for 2 to 4 weeks. For selected, more difficult cases, an implantable monitoring device named loop recorder may be used to record the ECG patterns during occasional but significant symptoms like syncope.

The system may be useful in several situations, among others:

- Detection of asymptomatic episodes of atrial fibrillation, which may require anticoagulation therapy to reduce the risk of stroke.
- Quick recognition of electrode lead failure, allowing fast intervention and avoiding inappropriate shocks.
- Reduction in the number of outpatient visits during long-term follow-up of patients with a pacemaker or implanted defibrillator.

### Heart failure (HF)^14,15^

Distant monitoring, or telemonitoring, is a promising strategy to improve the outcomes of HF treatment, allowing remote monitoring of patients so physicians can intervene early when evidence of clinical deterioration is present. The approaches vary from computerized systems for decision support to programs managed by nurses or physicians. A dedicated hardware or a smartphone may be used to transmit the patient's data (for example, symptoms, weight, blood pressure, and heart rate). A structured phone support, which can better guide the patient and offer specialized treatment to HF patients, has been shown to reduce the mortality and hospitalizations due to HF, improve quality of life, reduce the cost of treatment of prescriptions based on evidence, and improve the patients’ knowledge and their knowledge about self-treatment.
